# Authoritarianism today—a revised measurement model

**DOI:** 10.3389/fpsyg.2026.1620427

**Published:** 2026-09-10

**Authors:** Marius Dilling, Ayline Heller, Clara Schließler, Johannes Kiess, Elmar Brähler, Oliver Decker

**Affiliations:** 1 Else-Frenkel-Brunswik-Institute, University of Leipzig, Leipzig, Germany; 2 GESIS, Leibniz Institute for the Social Sciences, Mannheim, Germany; 3 Clinic and Polyclinic of Psychosomatic Medicine and Psychotherapy, Rostock University Medical Center, Rostock, Germany; 4 Department of Medical Psychology and Medical Sociology, University of Leipzig, Leipzig, Germany; 5 Sigmund Freud University, Berlin, Germany

**Keywords:** authoritarianism, confirmatory factor analysis, conspiracy mentality, conspirituality, critical theory, Germany, romanticism, superstition

## Abstract

**Introduction:**

In light of recent social developments, debates about the adequacy of Altemeyer’s widely-used scale on “right-wing authoritarianism” have (re-)gained momentum, as a shift in the articulation and expression of authoritarianism could be observed. In the German-speaking realm, researchers argue for a reconsideration of the original conceptualization of authoritarianism, drawing on a developmental-psychological model and a social-theoretical basis in the tradition of Critical Theory. Making this debate accessible to an international readership, we propose a measurement model that conceptualizes authoritarianism as a third-order factor comprising two distinct authoritarian psychodynamics — *sadomasochism* and *fetishism* — as second-order factors.

**Methods:**

After deriving these factors theoretically, we test the proposed model by means of confirmatory factor analysis on a German representative dataset (N = 2,466).

**Results:**

The proposed model fits the data very well (scaled CFI = 0.965, scaled RMSEA = 0.058, scaled SRMR = 0.044) with significant loadings throughout, though it cannot be statistically distinguished from two equivalent specifications and shows limited reliability at the higher-order level (*ω*l2 = 0.67).

**Discussion:**

By critically reconsidering the origins of authoritarianism and systematically operationalizing and testing our theoretical claims, the paper sheds light on the concept’s evolving manifestations and contributes to a nuanced understanding of authoritarian tendencies amidst emerging societal changes.

## 1 Introduction

Following early works by Fromm ([1936] 1987) and others, research on authoritarianism first gained momentum in the 1950s, shaped by the study *The Authoritarian Personality* that was conducted by the so-called Berkeley group around Adorno et al. (1950). Authoritarianism was thought to explain different types of resentment and prejudice as a result of a specific socialization in (late) capitalist society. The most prominent outcome of the studies was the Fascism-Scale (F-Scale), a questionnaire used to measure authoritarianism on nine distinct dimensions. Moreover, specific patterns of scores and distinct sociodemographic characteristics were used to establish different types of authoritarians, and qualitative interviews were conducted to gain insights into underlying psychodynamics.

Today, one of the most commonly used measures to operationalize authoritarianism is Altemeyer’s scale (Altemeyer, 1981, 1988, 1996) on “right-wing” authoritarianism (RWA). It reduces the concept to three dimensions, omitting both the developmental-psychological model as well as the social-theoretical basis. Early on, this reduction has been criticized both from a theoretical as well as a methodological standpoint (Duckitt and Sibley, 2009; Hopf, 1992; Oesterreich, 2005; Rippl et al., 2000). Schmidt (2023) even argues this development to be a degenerative process (following Imre Lakatos’ concept of a research program), as the six omitted scales could have been used to explain additional phenomena related to authoritarianism or they could have had different impacts on the same dependent variable. Furthermore, by leaving out the qualitative interviews that were part of the original study, and by not following a mixed-methods approach as advanced and promoted by Else Frenkel-Brunswik, the validity of the scales was reduced (Schmidt, 2023).^1^

It seems that this academic debate around both the concept itself and its measurement has taken different routes in the English- compared to the German-speaking realm. While English-speaking research has mainly focused on quantitative empirical analyses, e.g., the role of situational factors, like (perceived) threat (Duckitt and Fisher, 2003; Feldman and Weber, 2023; Osborne et al., 2023), German-speaking research has taken a different turn, explicitly reconsidering the original psychoanalytical and social-theoretical basis. Culminating in the protests against the COVID-19 pandemic, that were especially vivid in Germany and Austria, a seemingly new dynamic has become increasingly visible, calling for such a readjustment of the RWA concept: Social movements and protests that, on one hand, present themselves as pro-democratic, seemingly anti-authoritarian and defiant, while on the other hand showing anti-pluralistic and anti-liberal stances, strong rigidity, and pejorative resentment. At the same time, there was a pronounced tendency towards delusional, paranoid, and reality-denying elements that have already been described in the form of conspiracist and superstitious beliefs in classical authoritarianism research. In the wake of these political and social events in recent years, claims of a “new” authoritarianism (e.g., “libertarian authoritarianism”; cf. Amlinger and Nachtwey, 2024; “destructive authoritarianism” cf. King, 2021; “affirmative authoritarianism”; cf. Jäger, 2022) have been offered as solutions to manifesting changes in the expression of authoritarianism. Most of these propositions and theories, however, lack a clear operationalization or sufficient empirical support. Going back to theoretical and empirical considerations by Fromm ([1936] 1987) and Adorno et al. (1950), that already assumed an authoritarianism sui generis that is independent of the conscious political positioning of individuals and groups, we aim to rethink and empirically validate a revised model of authoritarianism.

Despite the outlined developments, we continue to assume that Altemeyer’s reduced three-dimensional scale, that is the foundation of most research on authoritarianism today, is a suitable operationalization of what Fromm ([1936] 1987) called a *sadomasochistic* psychodynamic - an affinity towards willing submission to the rule of a strong leader and a tendency to punish deviant behavior and those who are marked as outgroup while wanting to maintain the societal status quo. This conception is suitable for capturing many, but not all authoritarian phenomena, as outlined above. We thus assume that in addition to this *sadomasochistic* dynamic, a second authoritarian dynamic is at work, which is not new, but its (re-)consideration allows for a shift in focus depending on the societal-historical context. We call it the *fetishistic* dynamic, representing the reality-denying elements of authoritarianism. We operationalize this distinct psychodynamic by including another second-order factor in the model. Fetishism consists of two first-order factors: conspiracy mentality and superstition, both of which were included in the original conceptualization by Adorno et al. (1950). Authoritarianism can thus be operationalized using these two second-order factors, *sadomasochism* (aggression, submission, conventionalism) and *fetishism* (conspiracy mentality, superstition), eventually reflecting a third-order factor *authoritarianism*. This third-order factor itself is influenced by current and historical social conditions, thus affecting the expression of the second-order factors.

By operationalizing these psychodynamics as latent, higher-order factors, we go beyond what Adorno et al. (1950) were able to implement using statistical methods available at the time. The assumption of intervening constructs that are not directly measurable allows us to investigate indirect relationships between observed variables according to our theoretical considerations (cf. Schmidt, 2023). We thus recognize the complexity of psychoanalytic and Critical Theory, while still using statistical modeling approaches to empirically test our assumptions, thereby expanding and enriching the approaches put forth by Adorno et al. (1950).

We proceed as follows. First, we explicate our theoretical claims by revisiting the psychoanalytic foundations of early works on authoritarianism, and by presenting our justification for assuming a second psychodynamic to be at work. As psychoanalysis and Critical Theory have often been criticized for its lack of empirical support, we then empirically substantiate our theoretical considerations in a second step: we propose and test a revised measurement model of authoritarianism, using confirmatory factor analysis (CFA) on German representative data. Finally, we critically discuss our findings in light of social conditions and socio-political dynamics, and consider new perspectives that a Critical-Theoretical understanding of authoritarianism offers in the study of ambiguous and at first glance even anti-authoritarian social phenomena such as conspiracist and anti-vaccination movements. We will also discuss methodological and theoretical limitations and identify future directions and avenues for follow-up studies.

## 2 Authoritarianism and its psychodynamics

In terms of theory and data, this study is part of the Leipzig Studies on Authoritarianism, a biennial, probability-based survey of the German population on political attitudes, which has been running since 2002. The studies are rooted in the early research on authoritarianism, Critical Theory and its psychoanalytic basis, including Freud’s model of the psychic apparatus. This model is based on a concept of the unconscious and on three psychological instances that make up the psyche: the *id*, the unconscious part and seat of our instinctual needs; the *ego*, with which we encounter the world on a conscious and realistic level; and the *superego*, conceived among other things as the seat of conscience, which develops through the social norms and values transmitted by (parental) authority, which are internalized in the process of socialization. The development of the superego, as of the psychic apparatus in general, is thus closely linked to the prevailing social conditions. Critical Theory enforces the idea of a socially produced unconscious, connecting it with a profound critique of political economy and its consequences for human life practice. Human behavior, both on an individual as well as a societal level, is driven by unconscious desires.

Despite the rejection of central basic assumptions (i.e., the unconscious desires that are influenced by socialization experiences) by scholars outside of Critical Theory, the concept of authoritarianism experienced a broad reception, especially in social and political psychology, which continues to this day (Feldman and Weber, 2023; Six, 1997). We propose to revisit early works on authoritarianism by Horkheimer ([1936] 1987) as well as Adorno et al. (1950) to gain a deeper understanding of authoritarianism and its dynamics today. As we stated in the introduction, authoritarianism in its current conception (as proposed by Altemeyer) cannot explain all authoritarian phenomena, if it is reduced to a mere reaction to threatening situations in the familiar pattern of submission, aggression and conventionalism (which, following the early works of Fromm, we operationalize as the *sadomasochism* factor). This even applies to right-wing extremism, a phenomenon closely linked to authoritarianism (Heller et al., 2022): Authoritarianism should theoretically explain most, if not all expressions of right-wing extremism. However, a growing number of studies (Amlinger and Nachtwey, 2024; Dilling et al., 2023; Schließler et al., 2020) argue that certain characteristics of right-wing extremism, such as its inherent subversiveness and the use of anti-state codes, cannot be grasped by the current conception of authoritarianism, but should still be viewed as inherently authoritarian. For example, Imhoff and Decker (2013) point to the fact that most right-wing extremist groups do not want to maintain the status quo – as suggested by the conventionalism factor within the current conception. Moreover, the de facto rejection of existing (democratic) governments calls into question the dominance of authoritarian submission within these groups. On the basis of qualitative interviews with participants of the COVID-protests, Amlinger and Nachtwey (2024; see also Nachtwey and Heumann, 2019) were able to distinguish two types of what they called “libertarian,” which differ significantly in terms of their attitudes to the status quo and in the extent of their belief in conspiracies. One type could indeed be captured by the sadomasochistic dynamic of aggression, submission and conventionalism (they call these socially rather integrated persons “authoritarian innovators”), while the other type, the “regressive rebels” seem to go beyond that: they are characterized by disappointment with neoliberal promises of upward mobility and express fears of social decline. They deal with these fears and biographical problems through a pronounced belief in conspiracies (often in combination with esoteric practices and superstition). Unlike the innovators, they do not want to reform the social order, but openly rebel against it, calling into question existing social norms. Revisiting conspiracy mentality and superstition and their corresponding psychodynamic foundation could help grasp these seemingly contradictory characteristics of right-wing extremist subcultures and other authoritarian phenomena.

In contrast to many contemporary approaches in the English-speaking research, Smallpage et al. (2023) have recently proposed an integrative concept that reconnects such reality-denying attitudes with the theoretical work of Adorno et al. (1950): They refer to conspiracy thinking, pseudoscience, esotericism, and related belief systems as *romanticism*.^2^ In not treating conspiracy beliefs as isolated phenomena, Smallpage et al. (2023) emphasize their embeddedness within a broader latent construct: their concept of a “romantic” mindset as a shared latent factor of such beliefs closely echoes the integrative approach of *The Authoritarian Personality*. Notably, these beliefs had already been identified by Adorno et al. (1950) as symptomatic of a particularly narcissistic variant of the *Authoritarian Personality* and seem to be in close relationship with authoritarianism as a whole (as they are able to explain a large part of the variance in RWA and Social Dominance Orientation with their latent construct; Smallpage et al., 2023).

Adorno et al. (1950) used the phenomenon of superstition to suggest that there may be another psychodynamic of authoritarianism at work. In addition to Fromm’s conception of *sadomasochism*, that he conceived as an “ego weakness” and that we view as the basis of the current conception of authoritarianism, they describe the inner psychic movements behind superstition as an indication that “the ego has already ‘given up’“(Adorno et al., 1950, p. 236), because it is no longer trying to determine its own life: instead, individual responsibility shifts onto external forces, whether imagined as benevolent or malevolent. Whereas the sadomasochistic psychodynamic locates authority in external or internalized figures — parents, leaders, conscience, commands and prohibitions — this psychodynamic locates it in supposedly transcendent forces such as fate, the stars, nature, or hidden conspiratorial powers. The common denominator is the surrender of agency: in both cases, responsibility for one’s own life is relinquished and transferred to an external (or internalized) source of authority. The very existence of these forces implies that history and its catastrophes remain subject to intentional forces rather than contingency, providing relief from the feelings of powerlessness arising from the complexity and unpredictability of modernity. While Adorno et al. (1950) did not theorize this decidedly as a separate psychodynamic, we propose to understand it as a delusional form of authoritarianism, that seeks dissolution and fusion with a powerful entity, thereby regressing to an even earlier stage of human development and turning away from reality entirely (Chasseguet-Smirgel, 1987). We call this the *fetishist* psychodynamic. We will look at both these two psychodynamics in more detail below.

### 2.1 The sadomasochist psychodynamic

The foundations for research on authoritarianism were laid at the Institute for Social Research in Frankfurt, Germany (Horkheimer, [1936] 1987). With the help of Sigmund Freud’s psychoanalysis, authoritarianism was understood as the result of socialization, which begins in early childhood and produces patterns of conflict resolution (so-called defense mechanisms). Along with the reconstruction of prevailing socialization patterns, types of authoritarian reactions in groups were also described along prevailing conflict management strategies. Adorno et al. (1950, p. 749) referred to this preliminary work when they described the “critical typology” of authoritarianism as a “social function.” They located the emergence of this in the bourgeois patriarchal nuclear family at the beginning of the 20th century. The capitalist society of the time was characterized by the suppression of drives in an authoritarian manner and by the adaptation to repressive social norms, enforced by the authority of the father, who embodied the prevailing rigid norms and values within the family. If the child wanted to avoid punishment and deprivation of love, it had to submit to this paternal authority. Nevertheless, this submission always contained a voluntary, even pleasurable element as it brought about the hope of one day taking the father’s place. Following Freud, they argued that during child development, the commands and prohibitions of the father’s (external) authority are internalized and become the child’s inner authority, the *superego*.

Social life and society always require that impulses of the id cannot be acted out directly. This leads to a fundamental contradiction and ambivalence in the individual that is part of every socialization, but takes on different forms depending on the social context, as different social norms and taboos are internalized in different ways and thus part of the superego. At the beginning of the 20th century, the societal position of the patriarchal father was increasingly devalued, leading to a particularly rigid and punitive superego (Adorno et al., 1950, p. 753). As a result, the superego acts with more severity against the impulses of the id and ultimately suppresses these impulses without external pressure, repressing them from consciousness. This process necessarily leads to frustration and aggression, which, instead of being directed at the source (towards the repressive authority), are acted out elsewhere, namely *projected*^3^ onto those who are imagined to be weak or alien (Fromm, [1936] 1987).

This so called “oedipal”^4^ dynamic consists of submission to authority and aggression against the weaker and was already described by Fromm as a “sado-masochistic character structure” (Fromm, [1936] 1987, pp. 117–118). The result is an ego that is “weak,” as it is crushed between the demands of the rigid superego, the drive needs from the id and the demands of reality, which must be recognized. However, the internalization of (traditionally: paternal) authority in the form of an – albeit rigid – superego also at least potentially enables the individual to emancipate from parental norms. For only under the condition that the superego is a separate entity from the ego is it possible for people to differentiate between their own wishes and the expectations of others (not just their parents). The *fetishistic* psychodynamic, in contrast, is thought to regress to an even earlier stage of human development, as we will outline in the next section.

### 2.2 Psychodynamic of fetishism

The term *fetishism* was first used to describe an overvalued use of an object for sexual arousal. Freud, however, recognized a psychic mechanism underlying this sexual preference: the denial of reality. Reality is recognized to the extent that the fetishist is capable of action in it while at the same time radically denying it (Freud, [1927] 1991). The ethnopsychoanalyst Morgenthaler (1974) already recognized that fetishism can shape entire groups and cultures: It is then no longer an individual perversion, but a collective denial of unbearable aspects of the outside world.

Our approach is similar to Fromm’s: He did not understand *sadomasochism* as an individual sexual perversion in the everyday sense. By using the term in the political sphere, he was grasping a desire as a driving force of human behavior that gives rise to group dynamics: Based on their research, Fromm and the Berkeley group postulated that *sadomasochism* was prevalent in authoritarian groups and becomes dominant when the need for authoritative security grows in times of social crisis. In Fromm’s case, the desire leading to observable behavior was shaped by a need for submission (masochism) and the tolerance and even enjoyment of suffering of others (sadism). We proceed similarly: we do not understand *fetishism* as an individual perversion based on genital-sexual fulfillment. But we are interested in the desire that underlies *fetishism* that is also effective in the social: the denial of an unbearable reality. Thus, not just authoritarian individuals but also authoritarian groups can be differentiated according to which authoritarian need and conflict processing constitutes them.

As mentioned above and in the words of psychoanalysis, this *fetishistic* dynamic is a regression to an even earlier developmental stage compared to the oedipal, *sadomasochistic* dynamic: the search for protection from an overpowering object is a reaction that borrows from the early relationship between the primary caregiver (traditionally the mother) and the child, when mother and child were still one, and neither self/object differentiation nor the superego were established. As it is not recognized as a separate identity, the mother is perceived as both all-powerful (when it is present and nurturing the child) and overpowering and evil (when it is absent and the child is overcome by fear). In this early stage, the child does not have an integrated impression of the mother. Instead, her perception is split in an only-good, and only-evil mother, as the psychic capacities are not yet strong enough to bear the ambivalences of integrating these images. In this *fetishistic* authoritarian dynamic, individuals seek fusion with the all-powerful mother-imago, thus regressing to pre-oedipal rather than an oedipal state. In this state, the “ego and superego can no longer exercise their control” (Chasseguet-Smirgel, 1987, p. 84f., own translation).

It is a state of perceived inseparateness between mother and child, in which the psychic instances of the child are not yet developed. In this regression the so-called narcissistic illusion to be one with an omnipotent mother helps to deny the hurtful and offending reality of powerlessness and being separate, that every child must inevitably live through. To help the child deal with this loss, there are objects of transition (Winnicott, 1969), like blankets or stuffed animals which stand for that which has been lost, that help them deal with individuation, eventually resulting in an acknowledgement of the reality of separateness and reality as such. For adults regressing to this state (in confrontation with unbearable narcissistic insult in current society), however, we use the psychoanalytic term of a *fetish*: an object that is charged with the illusion of being omnipotent through total identification with the pre-oedipal mother (Smirnoff, 1972). In the child’s development, a detachment from the mother/child dyad succeeds through the recognition of a third party existing outside this dyad (traditionally the father). He thus also becomes the representation of all difference and the demands and restrictions existing in social reality. Hence, if the identification with a “maternal” authority is in the foreground, the reality principle is abandoned – which is traditionally enforced via identification with the father or other authorities – and the pleasure principle prevails. Here lies a main difference to the *sadomasochistic* dynamic: Just as the (individual) fetishist draws pleasure from the fantasy of unity with an almighty mother, the group enables the fantasy of a unity in which differences – and the narcissistic insult associated with difference – no longer have to be endured. Authoritarian groups can thus be differentiated according to which authoritarian need and conflict processing constitutes them.

Instead of recognizing the reality of authoritarian or paternal law (*sadomasochistic* dynamic), as identification with a (father-)authority demands, the world is shaped according to one’s own desires. So, while the psychodynamic of *sadomasochism* still enables having a desire for difference, *fetishism* is about “eliminating the difference to experience grandiosity” (Decker et al., 2020, p. 192, own translation). Any form of dependency and limitation, whether of one’s own abilities or from outside, is denied. Within the fetishistic dynamic, aggressions are thus a reaction to the threatening collapse of a phantasized reality – which is cultivated not only by the individual, but also at the group level – inflicted by the confrontation with an unbearable reality and the “others” on the outside (not a detour satisfaction of desires forbidden by the (father-)authority as in the *sadomasochistic* dynamic). It is exactly this delusionary, reality denying, paranoid-like mode, that we find in superstition but also in conspiracy mentality.

The paternal-oedipal desire for submission to an authority and aggression towards the ‘weak’ as characteristics of the *sadomasochistic* dynamic include the recognition of the reality represented by the authority. The maternal preoedipal *fetishistic* dynamic differs fundamentally in this respect: the authoritarian group, bound together, e.g., by a shared belief in a conspiracy mentality and/or superstition, is characterized by a denial of reality (Chasseguet-Smirgel, 1987) and the need to merge with an omnipotent authority or power as a fetish (reflecting the only-good mother; e.g., benevolent nature or stars). Anything outside this group denial is perceived as a threat and persecuted (reflecting the only-evil mother; e.g., in terms of a all powerful entities/conspiracies). Thus, the destructive impulse is also inherent in this psychodynamic.

Following Adorno et al. (1950), we assume that it is social conditions that lead to group dynamics and individual needs, such as the sadomasochistic and fetishistic psychodynamic and that periods of transformation and crisis make such group dynamics, and the psychological needs associated with them, visible in a particularly condensed way. This was the case in the 1930s when the Berkeley group described the psychodynamics of *sadomasochism* and the phenomena of superstition and conspiracy beliefs. And this is particularly evident today, for example, in the protests against the measures to contain the COVID-19 pandemic in Germany and Austria — where “classic” authoritarians, supporters of esoteric and superstitious beliefs as well as followers of conspiracy theories joined together with a seemingly contradictory self-image as anti-authoritarian and pro-democratic. A similar constellation can also be observed in authoritarian post-truth politics, most notably in Trumpism.

Thus, we distinguish between two psychodynamics measured at the individual level. However, they are not independent of group dynamics and current social conditions. In fact, similar psychological needs may lead individuals to form authoritarian groups — which, in turn, foster group dynamics — while these same psychodynamics and corresponding needs are influenced by prevailing social conditions experienced through the early and lifelong socialization process. In order to empirically test our assumptions about the unity of these concepts, defining a measurement model and testing it using confirmatory factor analyses is appropriate. We operationalize the assumed psychodynamics as second-order, latent factors leading to different expressions of authoritarianism (the first-order factors). Since we assume authoritarianism to be socially produced – in a Critical-Theoretical understanding, psychodynamics are the product of current social conditions – we assume that these psychodynamics are themselves influenced by a higher-order factor *authoritarianism*. Decker et al. (2020) found initial empirical evidence for this measurement model and in the following, we want to put this conceptualization to an empirical test using confirmatory factor analysis on a probability-based German national sample.

## 3 Sample and methods

### 3.1 Sample and participants

Data was collected in early 2022 (March to May) as a cross-sectional probability-based study. The sample was part of a multi-topic survey conducted by the independent polling institute USUMA in two parts: in the first part, sociodemographic data were collected by 193 trained interviewers; the second part was completed independently by the respondents. For randomization, Germany was initially divided into 258 sample points. The interviewers then selected households (6,192 households) using the random route method and determined the target persons using the Kish selection grid. Inclusion criteria included a minimum age of 16 and the ability to read and understand the German language. Informed consent was obtained by all participants, at least one parent and/or legal guardian was informed in case of minors. The response rate was 41.2% with 50.12% of respondents being female^5^. Average age of respondents was 49.25, 4% of respondents indicated that they were unemployed at the time of the survey, 24.66% had a university entrance qualification, and the average household equivalent income was 2,087 Euro. Detailed information on sociodemographic variables can be found in Table A1 in the Appendix. An oversampling of the Eastern German population was administered to ensure sufficiently large sample sizes for both parts of the formerly separated federal republic. Participants with missing information on any of the involved variables were deleted (listwise deletion, *N* = 56)^6^, leading to a final sample size of *N* = 2,466.

### 3.2 Measures

#### 3.2.1 Sadomasochism

Sadomasochism was assessed using the short scale authoritarianism (*Kurzkskala; Kurzskala Autoritarismus*; KSA-3, Beierlein et al., 2015; Heller et al., 2022). The KSA-3 scale consists of nine items measuring authoritarianism on the three dimensions proposed by Altemeyer (1981) with three items for each dimension: *authoritarian aggression*, a*uthoritarian submission*, and *conventionalism*. While the Aggression-Submission-Conventionalism Scale (ASC, Dunwoody and Funke, 2016) is mainly used in the US context, the KSA-3 allows for a shorter, similarly targeted assessment of Altemeyer’s concept, and it has been validated for the German context, where it is used in many large survey programs (Sorrentino et al., 2025). A five-point Likert-type scale reaching from 1 (strongly disagree) to 5 (strongly agree) is used for scoring.

#### 3.2.2 Fetishism

*Conspiracy mentality* was assessed using a selection of three items based on the *Conspiracy Mentality Scale* (Imhoff and Bruder, 2014; Imhoff and Decker, 2013) with a Likert-type response scale reaching from 1 (strongly disagree) to 7 (strongly agree) only labeled at the endpoints. Like the original five item scale and the more elaborate, 15-item *Generic Conspiracist Belief Scale* (Brotherton et al., 2013), it is intended to measure a general susceptibility to conspiracy theories in an efficient manner. It has been previously used in large scale sociological and political surveys in Germany. *Superstition* was assessed using four items with each item covering a specific superstition (i.e., the belief in lucky charms, healers, fortune tellers, and horoscopes). Respondents are asked to rate their belief of the respective statement on a four-point scale (“certainly not true”, “probably not true,” “probably true,” “certainly true”). While this scale has not been psychometrically validated, it has been regularly included in the German General Social Survey (ALLBUS; GESIS, 2019), a large, biennial survey on (political) attitudes and behaviors, that has been running since the 1980s and is also part of the International Social Survey Programme (ISSP). Item wording as well as descriptive statistics for every item are documented in Appendix-Table A2.

### 3.3 Statistical analyses

First, descriptive item statistics (mean, standard deviation, skewness, and kurtosis) and inter-item correlations using Spearman’s rank coefficient were calculated.

**Figure 1:**
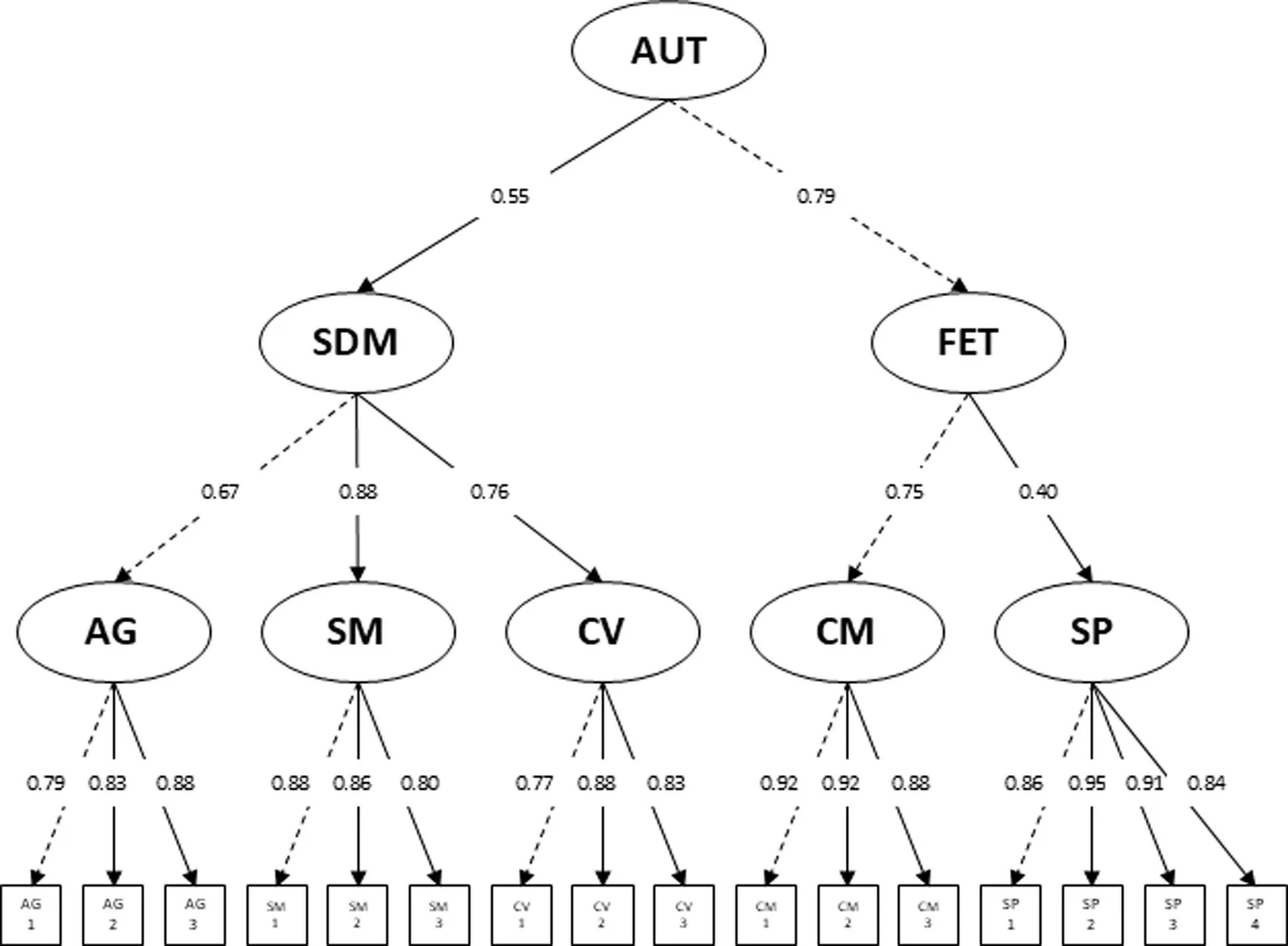
Structure tested in CFA and factor loadings for each dimension. Standardized factor loadings. AUT, third-order factor *authoritarianism*; SDM, second-order factor *sadomasochism*; FET, second-order factor *fetishism*; AG, first-order factor *authoritarian aggressio*n; SM, first-order factor *authoritarian submission*; CV, first-order factor *conventionalism*; CM, first-order factor *conspiracy mentality*; SP, first-order factor *superstition*. As indicated by the dashed lines in this figure, one item per first-order factor and one factor per higher-order factor are fixed to 1. Latent variance of the third-order factor is also fixed to 1 to ensure model identification. All loadings are statistically significant.

For the main analysis, we reexamined the factor structure proposed by Decker et al. (2020) (see Figure 1) using confirmatory factor analysis with the weighted least square mean and variance adjusted (WLSMV) estimator. We thus assume a superordinate third-order factor *authoritarianism* made up of second-order factors: *fetishism* and *sadomasochism*. *Fetishism*, in turn, is made up of the two first-order factors of *superstition* and *conspiracy mentality*, while *sadomasochism* consists of three dimensions: *authoritarian aggression*, *authoritarian submission*, and *conventionalism*. Factor loadings of the first item of each first-order factor as well as the factor loading of the second-order factor *fetishism* on the third-order *authoritarianism* factor were all fixed to 1. As higher-order factors can only be estimated with three indicators, the variance of the third-order factor was fixed to 1 as well.^7^ To assess model fit, we will report the following scaled fit indices: comparative fit index (CFI) and the Tucker-Lewis index (TLI) as well as the root mean square error of approximation (RMSEA) and finally the standardized [root] square residual (SRMR). Following Schermelleh-Engel et al. (2003) we will consider a CFI and a TLI of above 0.95 as an acceptable fit and above 0.97 for a good fit, while RMSEA and SRMR should fall below 0.08 to be acceptable and below 0.05 to indicate a good fit.

Based on the model, we then calculated multiple indicators for internal consistency. Choosing the right indicator for internal consistency in complex, higher-order factors has been a much-debated topic (Flora, 2020). While Cronbach’s Alpha is still the most widely used indicator, its assumption of (essential) tau-equivalence is rarely true and almost never tested. In addition to Cronbach’s Alpha, we will thus report McDonald’s ω McDonald, (1999), as it is based on the CFA and more suitable for multidimensional and higher-order models (Trizano-Hermosilla and Alvarado, 2016). In the case of *superstition*, we will also be reporting ordinal alpha, an indicator specifically designed for scales with ordinal indicators (Zumbo et al., 2007).^8^ For the second-order factors, we will be reporting ω_L1_ and ω_L2_ with the former describing the internal consistency of the second-order factor based on the variance at item level, and the latter capturing internal consistency of the second-order factor based on the variance of the first-order factors. Finally, reliability of the third-order factor is assessed using ω_L2_ only, as there is not yet an indicator available that allows for third-order structures to be tested on an item level especially when a mix of both ordinal and metric indicators is present. A mere calculation of internal consistency across all items would ignore the complexity of the model and is thus not fit as an indicator either.

RStudio 2023.12.1 with R version 4.2.2 as well as *STATA 16.1* was used for the analyses. Specifically, the package *semTools* was used for calculation of reliability indices, *lavaan* and *STATA 16.1* were used for the CFA, and *semPlot* was used to generate the graphical output.

## 4 Results

In order to empirically substantiate our decision to conceptualize *superstition* and *conspiracy mentality* as part of a complex authoritarian dynamic, we proceed as follows: We first report item and factor statistics on a descriptive level. We then move on to the CFA, where we will outline our identification strategy, report the factor loadings and then report the model fit. We conclude by presenting different reliability indices for the different levels of the measurement model.

### 4.1 Descriptive statistics

Results of descriptive item statistics and inter-item-correlations can be found in the Appendix-Tables A2 and A3. Correlations were generally high within the first-order constructs, and lower, but still significant within the second-order constructs. Correlations with the items of the *superstition* factor were lowest, and in some cases non-significant. We want to point out that low manifest correlations at the item level do not preclude the existence of higher factor structures, e.g., it is possible that certain items or first-level factors do not capture the higher-order factor as well as others, resulting in lower manifest correlations compared to the corresponding latent correlations (see, e.g., Brown, 2015, or Zick et al., 2008, as an empirical example). We thus move on to the main analysis.

### 4.2 Confirmatory factor analysis

Figure 1 shows the factor loadings for all indicators at the respective levels. All factor loadings are significant (*p* < 0.001). The first-order factor superstition shows the lowest loading of all (*λ* = 0.40) and is only moderately associated with the second-order factor *fetishism*. *Fetishism* shows a higher loading on the third-order factor (*authoritarianism*; loading *λ* = 0.79) than *sadomasochism* (*λ* = 0.55).

Regarding the model fit, the scaled CFI for the measurement model is 0.965, while scaled TLI lies at 0.957, both implying a good model fit. The scaled RMSEA for the measurement model is 0.058 (CI: 0.055–0.062, *p*-close < 0.001), which also implies an adequate model fit. Finally, the scaled SRMR of 0.044 presented here also corresponds to a good model fit. Overall, these results indicate a good model fit.

Alternative models with (1) two correlated second-order factors and no third-order factor or with (2) only one second-order factor *sadomasochism* and two separate first-order factors *conspiracy mentality* and *superstition* are statistically equivalent and thus yield the same model fit (Brown, 2015; Hoyle, 2023). They cannot be empirically differentiated. A model with just one first-order factor *sadomasochism* and no inclusion of *conspiracy mentality* and *superstition* is not nested and cannot be compared because information criteria are not available for models based on WLSMV-estimation yet. The same holds true for a bifactor model with a general factor *authoritarianism* and five orthogonal specific (group) factors, that yields an inadequate model fit and is incompatible with current theories of authoritarianism (see Appendix-Table A5 for fit indices of all models). A formative model was not tested as it is not identified within the covariance-based structural equation modelling approach (Riebel and Lichtenberg, 2023; see section 5.1. for a discussion of alternative models).

### 4.3 Reliability of the final model

Reliability indices for all dimensions can be found in Table 1. For the first-order factors, McDonald’s Omega and Cronbach’s Alpha ranged between 0.87 (*conventionalism*) and 0.93 (*conspiracy mentality*) pointing towards good internal consistency.

**Table 1:** Reliability measures of each dimension.

Level	Dimension	McDonald’s omega	Cronbach’s alpha (ordinal)	Omega Level 1	Omega Level 2
First-order	Authoritarian aggression	0.87	0.87		
	Authoritarian submission	0.88	0.88		
	Conventionalism	0.87	0.87		
	Conspiracy Mentality	0.93	0.93		
	Superstition	0.90	0.91 (0.94)		
Second-order	Sadomasochism			0.76	0.82
	Fetishism			0.52	0.58
Third-order	Authoritarianism			-	0.67

For the second-order factors, *sadomasochism* showed good values of internal consistency both at the item level (*ω*_L1_ = 0.76) as well as based on the variance of the first-order factors (*ω*_L2_ = 0.82). However, internal consistency of the *fetishism* factor was poor both at the item level (*ω*_L1_ = 0.52) as well as based on the variance of the first-order factors (*ω*_L2_ = 0.58). From a methodological standpoint this could be due to a large difference in response scales between *conspiracy mentality* and *superstition* (in the case of *ω*_L1_) or due to the low number of indicators (in the case of *ω*_L2_). Further reasons for these inadequate values are discussed in section 5.

Like the second-order factor *fetishism*, the internal consistency of *authoritarianism* is rather low (*ω*_L2_ = 0.67). As this value is based on two indicators only, it can still be viewed as acceptable.

## 5 Discussion and limitations

In light of recent social developments, debates about the adequacy of Altemeyer’s widely-used concept and scale on “right-wing authoritarianism” have (re-)gained momentum, as a shift in the articulation and expression of authoritarianism could be observed. In Germany and Austria, this was particularly evident in the anti-vaccine protests during the COVID-19 pandemic: At first glance, they may appear anti-authoritarian, since they see themselves as democratic resistance against an authoritarian, state-mandated vaccination campaign that targets individual freedom. In fact, they are composed of a milieu open to the far-right and steeped in conspiracy theories. Taken together with the rise of post-factual politics across the globe, the academic debate regarding the appropriateness of the internal structure of authoritarianism as a construct was (re-)opened, especially in German-speaking research. Making this debate accessible to an international readership, we revisited the original Critical-Theoretical conception of authoritarianism (Adorno et al., 1950; Fromm, [1936] 1987). Drawing from their work as well as theoretical considerations based on psychoanalytic social psychology, we propose a higher-order measurement model with two second-order factors, *sadomasochism* and *fetishism* bound together by a third-order factor *authoritarianism*. We understand *sadomasochism* as an oedipal dynamic consisting of submission to authority (masochism) and aggression against the weaker (sadism) while trying to maintain the social status quo. This dynamic was already described by Fromm ([1936] 1987) and the Berkeley group and connected to the specific family constellation of the time (Adorno et al., 1950). Psychoanalytically speaking, *fetishism* can be viewed as a pre-oedipal dynamic, and its expressions were also already considered to be part of the Authoritarian Personality (Adorno et al., 1950), without being theorized decidedly as a separate psychodynamic. Drawing from these traditions, we understand *fetishism* as the denial of an unbearable reality as it is expressed in conspiracist and superstitious beliefs.

The proposed model shows a very good global fit with significant factor loadings. In terms of factor loadings, our assumed third-order factor *authoritarianism* exhibits more shared variance with *fetishism* (*superstition* and *conspiracy mentality*) than with *sadomasochism*. These findings indicate that the proposed hierarchical model provides a plausible representation of the data. Given the statistical equivalence of several alternative specifications, however, the third-order factor is supported primarily by theoretical rather than empirical considerations.

While these results indicate that it is indeed fruitful to understand *conspiracy mentality* and *superstition* as parts of a distinct dynamic, that is itself part of *authoritarianism*, some major methodological challenges have to be pointed out and their theoretical implications need to be discussed. First, the third-order factor (*authoritarianism*) shows low reliability values (*ω*_L2_ = 0.67), which could in part be due to the small number of indicators for this third-order factor.^9^ It should be mentioned that this measurement model is not primarily aimed at providing an overall score, but rather at analyzing the relationships between the different dimensions of authoritarianism and their connections to social phenomena. We understand authoritarianism to be comprise two different psychodynamics, thus loosening the homogeneity assumption of the indicators in the higher-order structure. As the global fit indices are good, the low reliability of the overall factor thus seems subordinate.

Second, while the introduction of a third-order factor is theoretically (and empirically) feasible, providing evidence that this assumption increases the predictive power of the overall model was beyond the scope of this paper. Results on *romanticism* as introduced by Smallpage et al. (2023), a factor that is conceptually very close, if not identical to *fetishism*, suggest that assuming a second-order factor significantly increases the explained variance in social dominance orientation (specifically aggressive inequality). Future studies should investigate relative effects and residual uniqueness of the proposed model with regard to different types of prejudice and anti-democratic resentment.

Third, the reliability issue mentioned above also holds true for the second-order factor *fetishism*: While the low ω_L2_ could again be attributed to the limited number of indicators (two first-order factors), this argument does not hold [for] ω_L1_ that is calculated based on the indicators at item level. From a methodological standpoint, another (fourth) pitfall should be addressed here: the ordinal character of the *superstition* items. While there are ω-measures for ordinal indicators in non-hierarchical models (i.e., uni- or multidimensional constructs), these have not been applied to second- or third-order factors. As ω_L1_ is not fit for categorical input data, it cannot be meaningfully interpreted for the second-order factor *fetishism*. The low omega values of the second-order *fetishism* dimension may further contribute to the low ω_L2_ of the third-order factor. While we acknowledge that the observed reliability pattern also warrants caution regarding the discriminant validity of the fetishism dimension, we would argue this pattern to be more consistent with measurement-related limitations, given the overall good model fit.

Fifth, the first-order factor *superstition* showed a low factor loading with regard to the second-order factor fetishism, and the manifest correlations of the items with the indicator items of the other dimensions were also low. In addition to these methodological issues, it is also possible that our operationalization of superstition is not ideal; we measure the belief in very specific paranormal phenomena (e.g., ‘Good luck charms sometimes do bring good luck.’), while the other dimensions tend to refer to more abstract, general principles. One possible solution for subsequent research could be to measure superstitious beliefs on a more abstract level. It could also be insightful to consider other phenomena that are based on a similar moment of reality denial indicative for the *fetishism*-factor: esoteric beliefs and spirituality as phenomena distinguishable from mere superstition could be the subject for follow-up research (Amlinger and Nachtwey, 2024; Knasmüller et al., 2023; Schließler, 2024) as they tend to show aspects of reality denial as well. This idea is further substantiated by the results of Smallpage et al. (2023): not only could they show that their second-order factor *romanticism* showed high intercorrelations among the first-order factors conspiracy, pseudoscientific and paranormal beliefs. Using SEM, they also revealed a significant connection between RWA and *romanticism*. As *romanticism* and *fetishism* are conceptually very similar and RWA is the basis of sadomasochism, these results suggest that the low intercorrelations and factor loadings of our superstition factor may in fact be a methodological artefact of poor operationalization. The additional consideration of esoteric beliefs/spirituality in our model could further differentiate *fetishism*, possibly increasing its reliability and thus helping to better grasp the new dynamic we are proposing, both from a methodological as well as a theoretical standpoint.

Sixth, the model is not particularly parsimonious, i.e., overspecification may be a problem. Nevertheless, it is theoretically sound as it responds to current research and social dynamics. The complexity of the model, especially in terms of the many subdimensions of the authoritarian syndrome, can be considered justified given the multifaceted, ambivalent nature of the phenomenon. By operationalizing psychodynamics as latent factors that cannot be directly observed, but can be derived from a sound theoretical basis, it presents a methodological extension and elaboration on the works of Adorno et al. (1950).

### 5.1 Alternative modelling approaches and future directions

A validated measurement model may serve as a basis for several subsequent analyses that may help better grasp authoritarian phenomena. Person-centered approaches like latent profile analyses can be used to examine different types of authoritarians and the different social milieus they occur in. Results from these analyses may also shed new light on the nature of the construct. Dilling et al. (2025), for example, used latent profile analysis and identified two types of profiles: those scoring high or low on all first-order factors and exhibiting strong or almost no resentments against outgroups, and those that showed mixed proportions of these first-order factors.^10^ This suggests some degree of independence of the two proposed second-order factors or psychodynamics. From a data-driven perspective, exploring alternative modeling approaches might thus be reasonable (e.g., formative or bifactor models). These approaches do not necessarily align with our theoretical considerations though. Let us elaborate on this.

A formative approach would conceptualize authoritarianism not as a single latent factor influencing its subordinate factors, but rather as a composite of distinct (causal) indicators (first-order factors) – such as *authoritarian aggression*, *submissiveness*, *conventionalism*, *conspiracy mentality* and *superstition* – that together shape the overall construct. Causality would be reversed in this type of model: it is not the latent trait *authoritarianism* causing the *sadomasochistic* and *fetishistic* dynamic, but rather it is these dynamics that together lead to authoritarian individuals. A formative modeling approach might better align with some of the profiles identified by Dilling et al. (2025), which demonstrate diverse ‘alloys’ of authoritarian traits, suggesting that these components may independently contribute to different types of authoritarians. From a theoretical standpoint, reversing causality is problematic: we argued above that, from a developmental psychological perspective, *authoritarianism* causes the two psychodynamics in the first place. Nevertheless, rejecting a purely formative conceptualization does not imply that exclusively reflective models represent the only viable option. The present model already relaxes the strict homogeneity assumption typically associated with higher-order reflective measurement by conceptualizing authoritarianism as comprising two qualitatively distinct psychodynamic processes. This suggests that future research could benefit from exploring hybrid measurement models that combine reflective and formative relationships. Despite their potential to better capture complex multidimensional constructs, formative-reflective and reflective-formative hybrid models have received surprisingly little attention in the social sciences and may therefore constitute a promising avenue for future methodological and theoretical research.

A bifactor model would allow for a differentiation between variance that is due to a general factor and variance that is explained by the distinct dimensions and – as Flora (2020) shows – is a possibility to counter the not yet available *ω*-measures for ordinal data in higher order models. However, bifactor models assume first-order factors to be orthogonal, thus the combination of higher-order and bifactor structures is not possible. Moreover, the general factor (a factor that loads on all manifest variables) would be assumed to be uncorrelated with the specific factors (Flora, 2020; Yung et al., 1999). Not only would such a model fit the data substantially worse (see Appendix-Table A5), it also does not align with our considerations from a theoretical perspective, as we assume the lower order constructs to be related to each other as well as the general factor.

Coming from theory, we thus reject both a formative as well as a bifactor modeling approach: it is originally the social conditions, as experienced during the early and lifelong socialization process (and not measured in our current model), that give rise to individual authoritarianism. Authoritarianism then influences its different expressions as operationalized by the second-order factors. Individuals scoring high on *authoritarianism* as a third-order factor are more likely to fall victim to either a *sadomasochistic* or *fetishistic* dynamic, thus more likely agreeing with the items belonging to the first-order factors. Again, it is the social conditions that are essential to determining which dynamic is prevalent in a society.

We have to note that our proposed model cannot be statistically differentiated from two equivalent models: (1) a model with two correlated second-order factors and no third-order factor or (2) a model with only one second-order factor *sadomasochism* and two separate first-order factors *conspiracy mentality* and *superstition*. While our analysis thus does not contradict our theoretical assumptions, it does not *prove* the claim of a third-order structure either. It does, however, strengthen our theoretical assumptions, in that the model fit does not contradict them. If proven useful in future studies, our model can build the basis to investigate interactions between social conditions, group processes and individual psychodynamics to better understand authoritarianism today.

In addition to further validating and refining the measurement model, investigating the temporal and intercultural stability of authoritarianism is of interest: Authoritarianism from the perspective of Critical Theory is always in interdependence with the current social conditions.^11^ Therefore, it is possible that the factor structure and specific expression of authoritarianism is temporally and culturally variant. Measurement invariance analyses should thus be used to test whether the model is stable across time and cultures. If it is, age-period-cohort analyses could be avenue for future research, to investigate temporal changes in the expression of authoritarianism.

Critical Theory and psychoanalysis have often been criticized for their lack of (quantitative) empirical support. Their claims cannot easily be operationalized as they do not aim to provide testable hypotheses. This study can be seen as a first contribution to systematically (re-)linking psychoanalytic social psychology and quantitative social psychology. Using confirmatory factor analysis, we operationalized two distinct psychodynamics that we derived from theory as two latent (second-order) factors. The existence of these factors is in line with the empirical results: the model fit is good. Nonetheless, this approach does not confirm that the second-order factors actually reflect the psychodynamics we originally theorized, and other statistically equivalent models are possible, though not supported by theory. Returning to the mixed-method approach initially put forward by Adorno et al. (1950), especially by Else Frenkel-Brunswik, could help to further validate the measurement model by investigating the meaning of the second-order factors. In-depth hermeneutics on individual or group interviews have been used to substantiate the claim of different psychodynamics being at work when it comes to authoritarian individuals and groups (e.g., Knasmüller et al., 2023, investigating the connection of conspiracy mentality and esoteric/spiritual beliefs; Kaufhold, 2024, investigating different psychodynamics of antisemitism and racism). These approaches could also be used to shed light on the connection between social conditions, group dynamics, and individual expressions of authoritarianism. An integration of the results from these studies with quantitative approaches, like multilevel modeling to investigate context effects on authoritarianism, is central to grasp the complexity of this multifaceted phenomenon.

In summary, the proposed model represents an important step forward in the measurement of authoritarianism: by (re-)integrating fetishism, i.e., the aspect of reality denial, in authoritarian dynamics alongside the dynamics of mere submission, aggression and conventionalism we hope to reopen the debate about a theoretically driven measurement model of authoritarianism to an international audience. Further research should focus on validating the complex structure in different samples (in particular in cross-cultural analyses) and integrating esoteric/spiritual beliefs as additional dimensions. Furthermore, extending and exploring the model through alternative model specifications could lead to a deeper understanding of authoritarian tendencies and dynamics worldwide. Finally, returning to a mixed-method approach and integrating results from both qualitative and quantitative analyses (e.g., by using paired samples) is central to understanding the complexity of the phenomenon.

## Data Availability

The dataset analyzed in the current study was generated as a joint project of several different universities. Due to missing consent of all parties involved, we are unable to make the dataset publicly available. The parts supporting the findings of this study will be provided by the corresponding author upon reasonable request.

## Footnotes

1 Altemeyer proposed a one-dimensional construct with three aspects that could not be reproduced empirically (cf. Funke, 2005). Following Altemeyer’s proposition, subsequent research focused on adequately capturing the three aspects while eliminating item overlap with outcome variables and reducing possible response biases by using balanced scales (cf. Heller et al., 2020; Schmidt, 2023).

2 Another comparable approach is discussed under the term conspirituality: the co-occurrence of conspiracy beliefs and (esoteric) superstition (Asprem and Dyrendal, 2015; Ward and Voas, 2011).

3 This projection can be viewed as a defense mechanism that is observable both at the individual as well as the group level.

4 It is described as an “oedipal” dynamic, referring to a development phase in which the dyadic relationship between primary caregiver and child has already been widened by a third object, traditionally the father, and the differentiation between the self and others has already been established.

5 Four respondents that stated diverse as their gender were excluded from the analysis.

6 Because the missing values for each indicator do not exceed 5%, we assume that exclusion did not lead to a large bias (Schafer and Graham, 2002).

7 Alternative modelling approaches were considered and will be discussed; however, several competing models are statistically equivalent, limiting their empirical discriminability (see section 4.2 and Appendix-Table A5).

8 Simulation studies by Rhemtulla et al. (2012) showed that items with less than five response categories should be treated as ordinal instead of metric.

9 Like Cronbach’s Alpha, McDonald’s Omega depends on the number of indicators. As mentioned above, ω_L2_ is calculated based on the variance of the second-order factors and is thus only based on two indicators.

10 The types of Amlinger and Nachtwey (2024; see also Nachtwey and Heumann, 2019), which are based on qualitative interviews, point in a similar direction.

11 The very focus on the sadomasochistic character structure as a result of rigid and authoritarian childcare in the Weimar Republic – that is, a specific era in German history – with a father who “hit” at its center, was a central point of criticism of the classic studies, since the generalizability to other contexts was questioned.
